# Prevalence, clustering and combined effects of lifestyle behaviours and their association with health after retirement age in a prospective cohort study, the Nord-Trøndelag Health Study, Norway

**DOI:** 10.1186/s12889-020-08993-y

**Published:** 2020-06-10

**Authors:** Siri H. Storeng, Erik R. Sund, Steinar Krokstad

**Affiliations:** 1 Department of Public Health and Nursing, Faculty of Medicine and Health Sciences, Norwegian University of Science and Technology, NTNU, Post box 8905, Håkon Jarls gate 11, N-7491 Trondheim, Norway; 2 HUNT Research Centre, Department of Public Health and Nursing, Faculty of Medicine and Health Sciences, Norwegian University of Science and Technology, NTNU, Levanger, Norway; 3 Faculty of Nursing and Health Sciences, Nord University, Levanger, Norway; 4 Levanger Hospital, Nord-Trøndelag Hospital Trust, Levanger, Norway

**Keywords:** Epidemiology, Lifestyle behaviours, Retirement age, Norway, HUNT

## Abstract

**Background:**

Lifestyle behaviours are potential risk factors for disease and mortality, but less is known about the association with health in retirement age. The aim of this paper was to study the prevalence, clustering and combined effects of lifestyle behaviours and their association with health outcomes in the first decade after retirement in a Norwegian cohort.

**Methods:**

Participants were 55–64-year-olds at baseline in the Nord-Trøndelag Health Survey 2 (HUNT2, 1995–97) who also participated in HUNT3 (2006–08). Logistic regression analyses were used to investigate the association of daily smoking, physical inactivity, risky alcohol consumption, disturbed sleep duration, excessive sitting time and low social participation before retirement with self-rated health (*n* = 4022), life satisfaction (*n* = 5134), anxiety (*n* = 4461) and depression (*n* = 5083) after retirement, 11 years later.

**Results:**

Low social participation and physical inactivity were the most prevalent lifestyle behaviours (41.1 and 40.6%). Risky alcohol consumption and disturbed sleep were the lifestyle behaviours most strongly associated with poor self-rated health, poor life satisfaction and anxiety after retirement (OR’s = 1.39–1.92). Physical inactivity was additionally associated with depression (OR = 1.44 (1.12–1.85)). Physical inactivity had the largest population attributable fractions for reducing poor self-rated health and depression (14.9 and 8.8%). An increasing number of lifestyle risk behaviours incrementally increased the risk for the adverse health outcomes.

**Conclusions:**

Risky alcohol consumption and disturbed sleep duration were most strongly associated with poor health outcomes after retirement age. On a population level, increased physical activity before retirement had the largest potential for reducing adverse health outcomes after retirement age.

## Background

In Norway, life expectancy is increasing along with a rising number of elderly [1]. A solution to combat an increasing dependency ratio (ratio of the dependent over the productive part of the working force) is to keep people longer in the workforce [2]. However, poor health increases risk of unemployment, disability pensioning and early retirement [3]. Oppositely, good health is a prerequisite for working into old age [4]. Retirement is a large transition period in people’s lives, but the effect of retirement on health remains unsettled [5, 6]. High demands and low job control have been found to increase risk of disability pensions [6–9]. Health effects of retirement may also differ between socioeconomic groups [10].

Lifestyle behaviours are important risk factors for non-communicable diseases, poor health and mortality [11]. Lifestyle behaviours have been found to cluster or co-exist [12, 13] and tend to change around retirement [14, 15]. A healthy lifestyle in midlife is associated with longevity [16] and the (pre-) retirement period may offer an opportunity to adopt healthier lifestyles [14]. Smoking, physical inactivity, high alcohol consumption and an unhealthy diet are often referred to as the “big four” potential modifiable risk factors influencing health and mortality [11]. In Norway, prevalence of daily smoking has decreased to 9% in 2019 [17]. Alcohol consumption in Norway has been increasing, especially in older age groups [18]. Further, increased sitting time, low social participation and disturbed sleep are emerging risk factors influencing health and mortality [19–31]. However, little is known about the importance of different lifestyle behaviours in working age on health in early years after retirement.

The aim of this study was to study the prevalence, clustering and combined effect of lifestyle behaviours and their association with self-rated health, life satisfaction, anxiety and depression in the first decade of retirement age, by using the Nord-Trøndelag Health Studies; HUNT2 (1995–97) and HUNT3 (2006–08) in a prospective cohort study.

## Methods

### Population

**Fig. 1:**
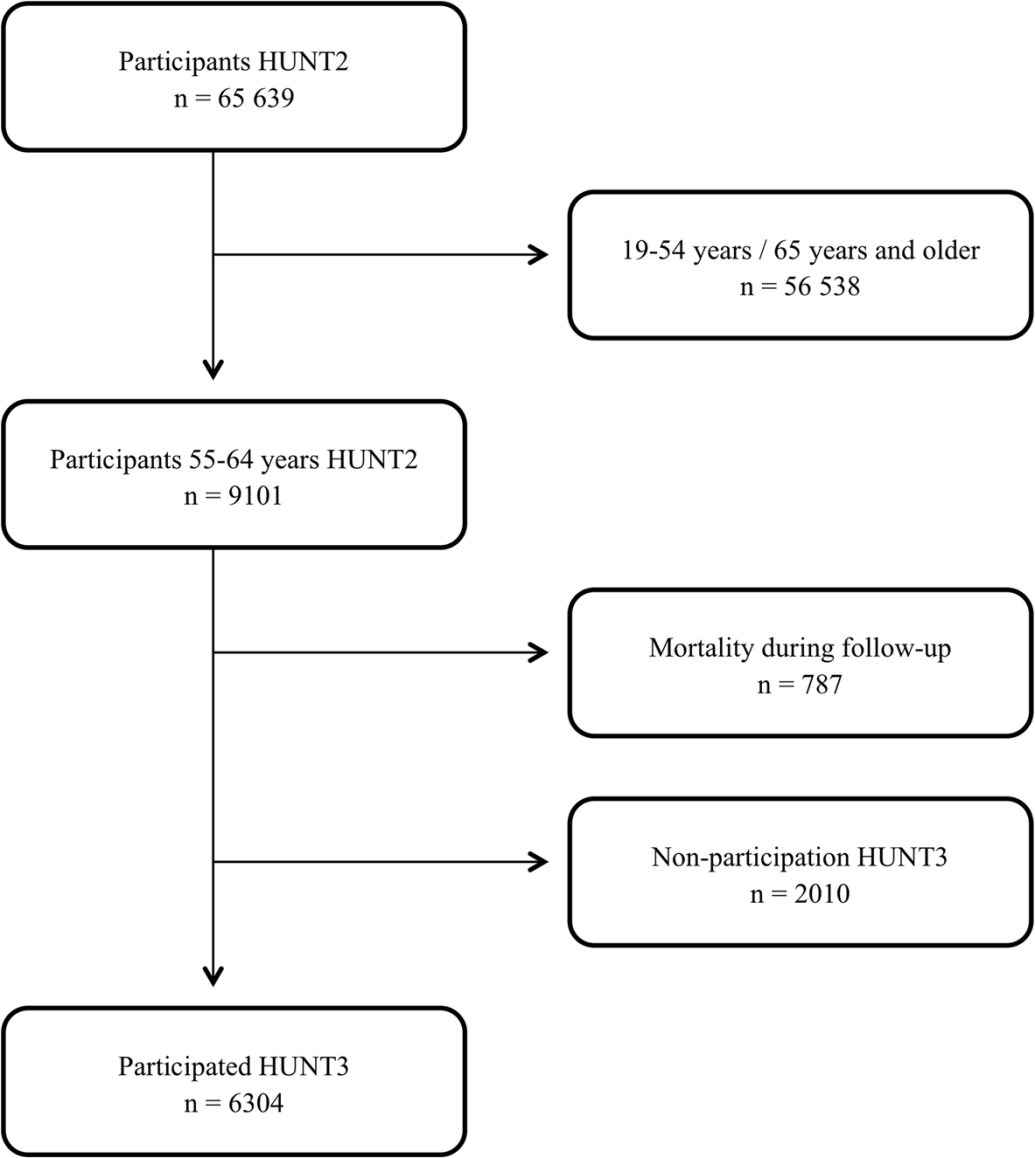
Flow chart showing the selection of participants

The selection of participants is shown in Fig. 1. The HUNT Study is a total county population-based study conducted with 11-year intervals (1984–86, 1995–97 and 2006–08) [32, 33]. All inhabitants in the county of Nord-Trøndelag in Norway aged 20 and older were invited to participate. Participants undertook a clinical screening test, blood sample collection and completed questionnaires. The overall participation rate in HUNT2 and HUNT3 was 69.5 and 54.1%, but higher among the middle aged (up to 85.5%) [32, 33]. All participants signed a written consent form to participate in the HUNT Study and it was approved by the Regional Committee for Medical Research Ethics. The material in this study was 55–64-year-olds reporting good self-rated health (*n* = 4022) / good life satisfaction (*n* = 5134) / no anxiety (*n* = 4461) / no depression (*n* = 5083) at baseline in HUNT2 who also completed HUNT3 (65–77 years), with an average of 11 years follow-up time. 65 years was used as a cut-off as it corresponds to the average retirement age in Norway [34].

### Measures

The lifestyle behaviours in HUNT2 were physical inactivity, daily smoking, risky alcohol consumption, prolonged sitting time, disturbance sleep duration and low social participation before retirement age (55–64 years). The lifestyle behaviours were selected based on previous research on their association with the studied health outcomes (summarised in Additional file 1). Questionnaires were developed by HUNT. Question texts, answer categories and coding of the independent variables are provided in Additional file 2. The cut-off for each lifestyle behaviour variable was based on available literature on their associations with health and mortality [19–21, 28, 35–40]. Unfortunately, we did not have enough information to include diet as a variable.

The outcome measures in HUNT3 were self-rated health, life satisfaction, anxiety and depression in the first decade after retirement age. The wording of self-rated health in the HUNT Study was *“How is your health at the moment?”* The original four answer categories *“very good” “good” “poor”* and *“very poor”* were dichotomised into *“good”* and *“poor.”* The question about life satisfaction was; *“Thinking about your life at the moment, would you say that you by and large are satisfied with life, or are you mostly dissatisfied?”* It had seven answer categories ranging from *“very satisfied” to “very dissatisfied”.* The first three and the last four answer categories were combined into *“satisfied”* and *“dissatisfied.”* Anxiety and depression were measured using the Hospital Anxiety and Depression Score (HADS), consisting of a separate score for anxiety (HADS-A) and depression (HADS-D). The scale has been validated and shows high sensitivity and specificity for scores ≥ 8 [41].

### Statistical analysis

Prevalence estimates were calculated for the 12 most common lifestyle risk behaviours and combinations of these. Logistic regression models were used to assess lifestyle risk behaviours’ association with self-rated health, life satisfaction, anxiety and depression in early years after retirement age. Population attributable fraction (PAF) was calculated for each lifestyle behaviour. PAF is the proportion of all cases that could be prevented by eliminating a specific risk factor. Lastly, logistic regression models were used to estimate the joint effect of increasing number of lifestyle risk behaviours on self-rated health, life satisfaction, anxiety and depression in early years after retirement. The models were adjusted for basic socio-demographic confounders (age, sex, education and marital status) and chronic illness. Non-participation in HUNT3 and mortality during follow-up from HUNT2 to HUNT3 could have introduced potential bias. This was evaluated in sensitivity analyses by including these competing outcomes in multinomial logistic regression models. Participants missing information on the relevant lifestyle variable in HUNT2 were excluded from that analysis. Odds ratios (OR) with 95% confidence intervals (95% CI) were reported. Analyses were carried out in Stata SE version 14 [42].

## Results

Table 1 shows descriptive statistics for 55–64-year-olds in HUNT2 who also participated in HUNT3 (*n* = 6304), with an average of 11 years follow-up time. The most prevalent lifestyle risk behaviours in HUNT2 were low social participation, physical inactivity and prolonged sitting time (41.1, 40.6 and 26.0%, respectively). 787 participants in HUNT2 died before the start of HUNT3 (October 2006) and 2010 participated in HUNT2 but not in HUNT3. There was a high percentage missing on all the lifestyle behaviours (9.4–26.8%), except for daily smoking (1.2%). Few reported risky alcohol consumptions measured by CAGE ≥ 2 (4.5%). Missing on the outcome variables varied from 1.6–15.5%.

**Table 1:** Descriptive statistics for 55–64-year-olds in the Nord-Trøndelag Health Study, HUNT2 (1995–97, baseline) and HUNT3 (2006–08, outcome), *n* = 6304

Lifestyle variables (HUNT2)		Adjustment variables (HUNT2)		Outcomes (HUNT3)	
	n (%)		n (%)		n (%)
Daily smoking		Sex		Self-rated health	
No	4779 (75.8)	Women	3362 (53.3)	Good	3899 (61.9)
Yes	1450 (23.0)	Men	2942 (46.7)	Poor	2159 (34.3)
Missing	75 (1.2)	Missing	0 (0)	Missing	246 (3.9)
Physical activity		Marital status		Life satisfaction	
Active	3154 (50.0)	Married	5196 (82.4)	Satisfied	5649 (89.6)
Inactive	2558 (40.6)	Not married	1102 (17.5)	Dissatisfied	554 (8.8)
Missing	592 (9.4)	Missing	6 (0.1)	Missing	101 (1.6)
Sitting time		Education		Anxiety	
≤ 7 h	3553 (56.4)	Primary	2269 (36.0)	HADS-A ≤ 7	4704 (74.6)
≥ 8 h	1638 (26.0)	Secondary	3171 (50.3)	HADS-A ≥ 8	623 (9.9)
Missing	1113 (17.7)	Tertiary	858 (13.6)	Missing	977 (15.5)
		Missing	6 (0.1)		
Alcohol		Chronic illness		Depression	
CAGE ≤ 1	4333 (68.7)	Yes	2690 (42.7)	HADS-D ≤ 7	4813 (76.4)
CAGE ≥ 2	283 (4.5)	No	3333 (52.9)	HADS-D ≥ 8	611 (9.7)
Missing	1688 (26.8)	Missing	281 (4.5)	Missing	880 (14.0)
Social participation				Mortality during follow-up	787
Participates	2978 (47.2)				
Seldom, never	2591 (41.1)			Non-participation HUNT3	2797
Missing	735 (11.7)				
Sleep duration					
7–9 h	4750 (75.4)				
≤ 6/ ≥ 10 h	767 (12.2)				
Missing	787 (12.5)				

*Abbreviations* used in the table: *CAGE* Screening questionnaire for risky alcohol consumption, *HADS* Hospital Anxiety and Depression Scale, *HADS-A* HADS-Anxiety, *HADS-D* HADS-Depression, *HUNT* The Nord-Trøndelag Health Study

The most prevalent lifestyle risk behaviour combinations when none of the lifestyle variables were missing are shown in Table 2. The most prevalent lifestyle risk behaviours were low social participation, physical inactivity and excessive sitting time. 50% of the participants reported none or a combination of these lifestyle risk behaviours. Physical inactivity and low social participation remained the most prevalent lifestyle risk behaviours when including all 55–64-year-old participants in HUNT2 and missing on lifestyle behaviours equals “no” (*n* = 9101, Additional file 3). Risky alcohol consumption and disturbed sleep were not among the 12 most prevalent combinations in either of the two analyses.

**Table 2:** The 12 most prevalent combinations of lifestyle risk behaviours in the Nord-Trøndelag Health Study (HUNT2, 1995–97), *n* = 5215

Combination	Prevalence (%)	Cumulative %	Smoking	Alcohol	Sitting	Inactive	Social	Sleep
1	635 (12.2)	12.2	–	–	–	–	–	–
2	511 (9.8)	22.0	–	–	–	–	+	–
3	438 (8.4)	30.4	–	–	–	+	–	–
4	408 (7.8)	38.2	–	–	+	–	–	–
5	403 (7.8)	46.0	–	–	–	+	+	–
6	246 (4.7)	50.6	–	–	+	–	+	–
7	238 (4.6)	55.2	+	–	–	+	+	–
8	228 (4.4)	59.6	+	–	–	–	+	–
9	209 (4.0)	63.6	–	–	+	+	–	–
10	195 (3.7)	67.3	–	–	+	+	+	–
11	160 (3.1)	70.4	+	–	–	–	–	–
12	140 (2.7)	73.1	+	–	–	+	–	–
Total	3811 (73.1)							

**Fig. 2:**
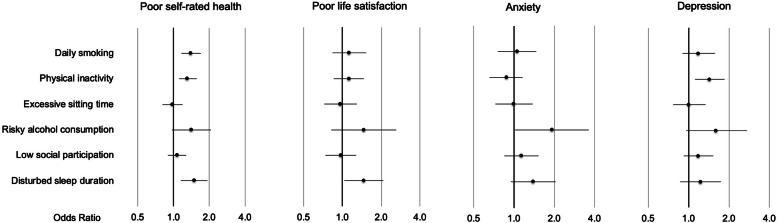
Odds ratios for poor health, poor life satisfaction, anxiety and depression

The associations between lifestyle risk behaviours and self-rated health, life satisfaction, anxiety and depression after retirement age are shown in forest plots in Fig. 2. Corresponding odds ratios and 95% confidence intervals from adjusted and unadjusted logistic regression analyses are provided in Additional files 4 and 5. Disturbed sleep duration, risky alcohol consumption, daily smoking, and physical inactivity were the lifestyle behaviours most strongly associated with poor self-rated health (OR’s ranging from 1.31–1.49). Disturbed sleep duration and risky alcohol consumption were most strongly associated with poor life satisfaction (OR = 1.47 (0.82–2.62) and 1.47 (1.04–2.08)). Risky alcohol consumption was the lifestyle risk behaviour most strongly associated with anxiety (OR = 1.92 (1.02–3.62)), followed by disturbed sleep duration (OR = 1.39 (0.95–2.04)). Physical inactivity showed an inverse association with anxiety after retirement age (OR = 0.88 (0.66–1.16)). Risky alcohol consumption and physical inactivity were most strongly associated with depression (OR = 1.61 (0.96–2.72) and 1.44 (1.12–1.85)). Low social participation and excessive sitting time had weak associations with the adverse health outcomes after retirement age in these analyses.

**Fig. 3:**
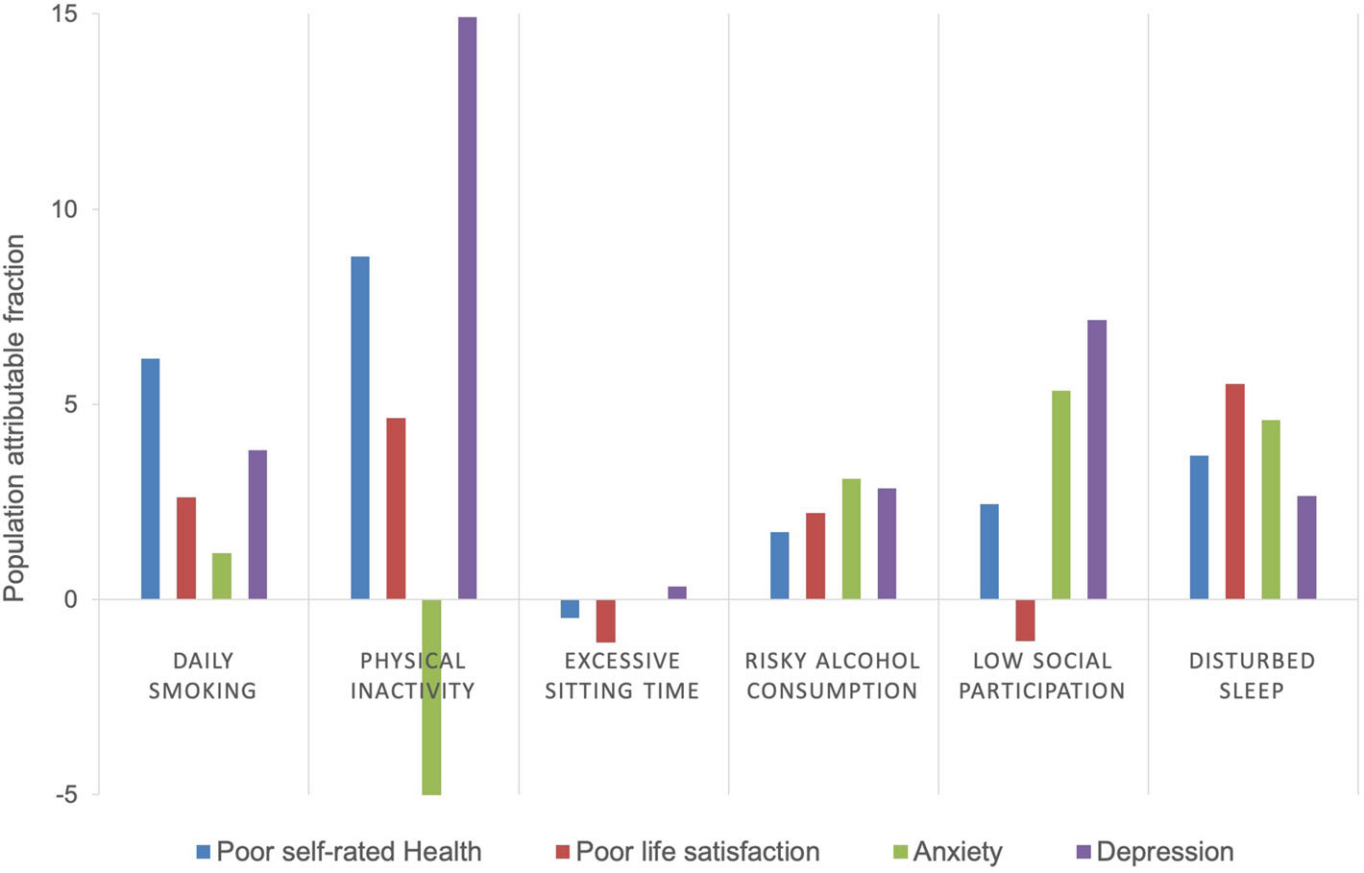
The proportion of all cases attributed to the lifestyle risk behaviours (population attributable fraction)

PAF for each lifestyle risk behaviour is shown in Fig. 3 and included in Additional file 4. Increased physical activity could theoretically have reduced the incidence of depression, self-rated health and poor life satisfaction in this population with 14.9, 8.8 and 4.7%. Physical activity had a potential adverse effect on the incidence of anxiety (PAF −5.8%). Increased social participation could potentially have reduced the incidence of depression and anxiety with 7.2 and 5.4%. Smoking cessation could have reduced the incidence of poor self-rated health in this population with 6.2%. PAF for reduced sitting time was small or negligible for all outcomes in this study population (PAF −1.1–0.34%). An increasing number of combined lifestyle risk behaviours incrementally increased the risk for negative health outcomes after retirement age (Additional file 6). Having four or more risk factors increased the risk of poor self-rated health with OR = 2.39 (1.60–3.58).

Non-participation in HUNT3 and mortality during follow-up from HUNT2 to HUNT3 were potential competing outcomes to the health outcomes in HUNT3. This was evaluated in sensitivity analyses by using multinomial logistic regression models (Additional files 7–10). Daily smoking and low social participation before retirement were more strongly associated with non-participation in HUNT3 and mortality during follow-up, compared to the health outcomes in HUNT3. Risky alcohol consumption and disturbed sleep duration were more strongly associated with the adverse health outcomes after retirement age (except for depression), compared to non-participation in HUNT3 and mortality during follow-up.

## Discussion

Low social participation, physical inactivity, excessive sitting time and combinations of these were the most prevalent lifestyle risk behaviours among 55–64-year-olds in HUNT2 in 1995–97. Risky alcohol consumption and disturbed sleep duration were less prevalent, but most strongly associated with poor self-rated health, poor life satisfaction and anxiety after retirement age. Physical inactivity was additionally associated with depression. Excessive sitting time and low social participation did not seem to be associated with the health outcomes measured after retirement age. At a population level, increased physical activity before retirement had the largest potential for reduction in the adverse health outcomes in early years after retirement. An increasing number of lifestyle risk behaviours incrementally increased the risk for the adverse health outcomes.

The main limitations of this study were the falling participation rate in the HUNT Study, healthy survivor bias and missing data on some of the lifestyle variables. Non-participants in HUNT3 have been found to have lower socioeconomic status, higher prevalence of several chronic diseases and higher mortality compared to participants [43]. Thus, the results from this study may be generalised to the healthier part of non-institutionalised older adults in populations comparable to the Norwegian setting.

Potential bias from loss of follow-up from HUNT2 to HUNT3 and mortality during the follow-up period was evaluated in sensitivity analyses where these competing outcomes were included in multinomial logistic regression models. Daily smoking and low social participation were more strongly associated with non-participation in HUNT3 and mortality during follow-up compared with the health outcomes. Risky alcohol consumption and disturbed sleep duration were more strongly associated with adverse health outcomes (except for depression) compared with non-participation in HUNT3 and mortality during follow-up. Thus, these lifestyle risk behaviours might be more important determinants for health compared with mortality in early years after retirement age. There was a high percentage missing on the questions regarding physical activity and risky alcohol consumption (measured by CAGE) in HUNT2, making the estimates less certain. Regretfully we did not have enough information on diet to include this as a lifestyle risk behaviour, which would have been relevant in this study of multiple lifestyle behaviours.

The most prevalent lifestyle risk behaviours among 55–64-year-olds in HUNT2 (1995–97) were low social participation (41.1%), physical inactivity (40.6%) and excessive sitting time (26.0%). A systematic review from 2015 identified common patterns of co-occurring health behaviours [12]. These were 1) no lifestyle risk behaviours, 2) daily smoking combined with alcohol, and 3) having all “big four” risk behaviours; smoking, poor nutrition, excess alcohol and physical inactivity. Another systematic review from 2016 also found daily smoking and alcohol to be the most prevalent risk behaviour combination [13]. This was followed by physical inactivity and unhealthy diet, while socioeconomic status was the strongest predictor for engaging in multiple lifestyle risk behaviours [13].

In this study daily smoking was not among the most common lifestyle risk behaviour combinations. In Norway, prevalence of daily smoking was 29–31% in 1995–1997 [17]. Since smoking prevalence has been found to be higher among non-participants in the HUNT Study [43], in addition to 1.2% missing on the questionnaire (Table 1), smoking prevalence in HUNT2 might be underestimated. Prevalence of risky alcohol consumption doubled from HUNT2 (1995–97) to HUNT3 (2006–08) for men and women over 60 years [18]. 28.2% participants aged 65 years and older reported drinking alcohol once or more weekly in HUNT3 [44]. In 2019 38% of 45–66-year-old Norwegians reported drinking alcohol once or more weekly; higher among older adults and men compared with women [45]. Thus, this lifestyle risk behaviour is most likely more prevalent today. There were few and possibly extreme observations in the category CAGE ≥ 2 in HUNT2, which may have underestimated the prevalence of risky alcohol consumption. Disturbed sleep duration, low social participation and excessive sitting time are seldom included in studies of multiple lifestyle risk behaviours [12, 13], making comparison difficult.

In this study, risky alcohol consumption, physical inactivity and disturbed sleep duration were the lifestyle risk behaviours most strongly associated with adverse health outcomes after retirement age. This is in line with previous findings showing associations between these risk behaviours and the studied health outcomes (summarised in Additional file 1) [26, 27, 29, 30, 46–58]. We found no or small associations between excessive sitting time and low social participation before retirement age with self-rated health, life satisfaction, anxiety and depression after retirement age. Previous studies have found associations between sedentary time [23–25, 36, 59–61], low social participation [31, 62–65] and the studied health outcomes. However, drawbacks such as large heterogeneity in the studies, lack of substantial evidence and methodological weaknesses in available studies are highlighted [66, 67]. Sedentary behaviour and low social participation are complex concepts that include several domains [31, 68], and the lack of standardised definitions and operationalisations hamper comparability between studies [12]. Lastly, causal pathways and directionality remain uncertain [36, 66, 67, 69].

Estimating PAF is important for guiding health policies. In this study, increasing physical activity before retirement showed the largest potential for reducing incidence of the adverse health outcomes after retirement age. This excludes anxiety where physical inactivity before retirement had an adverse effect (PAF −5.8%). A study based on HUNT1 and HUNT2 found 12% PAF for physical activity on the incidence of depression in the adult population, regardless of intensity [70]. The deviation from our PAF value (14.9%) could be explained by differing time periods, age groups and cut-offs for physical activity. Worldwide, it is reported 6–10% incidence reduction of non-communicable diseases by engaging in physical activity [71], in addition to a substantial global economic advantage [72]. The study based on HUNT data also reported an inverse relationship between physical activity and anxiety [70], similar to our results. In systematic reviews there has been found positive associations between physical inactivity and anxiety [23, 56, 57], but the directionality of the relationship remains unsettled [23, 57, 73]. Physical activity has been found to improve anxiety symptoms in intervention studies on healthy adults [56, 74], but aerobic exercise has not been found to be effective in treatment of clinical cases of anxiety [75]. Thus, the type of physical activity and severity of anxiety could play a role.

Risky alcohol consumption was strongly associated with the studied adverse health outcomes (OR 1.41–1.92). However, the prevalence and potential reduction in incidence of the studied adverse health outcomes by eliminating this risk behaviour was low (PAF 1.7–3.1%). Low social participation had the largest PAF for depression and anxiety (PAF 7.2 and 5.4%), and thus the highest potential for improvement in mental health. Smoking cessation could potentially reduce the incidence of poor self-rated health, depression and anxiety with 6.2, 3.8 and 2.6%. The prevalence of daily smokers in Norway decreased from 33% 1995–1997 to 9% in 2019 [17]. Thus, public health gains from reducing this lifestyle risk behaviour may already have been obtained. Reduced sitting time did not seem to have potential to affect the incidence of the adverse health outcomes included in this study (PAF −1.1–0.34%). An increasing number of lifestyle risk behaviours incrementally increased the risk for adverse health outcomes. A few studies have found that engaging in multiple lifestyles risk behaviours is associated with poor self-rated health [76–78] lower health-related quality of life [79] more anxiety [80] and depression [80, 81]. And combined effects of lifestyle behaviours have been shown to affect all-cause mortality [16, 82].

## Conclusions

The positive effects of adopting a healthy lifestyle around pensioning age presents opportunities for implementing interventions to enable people to work longer. Adopting or maintaining a healthy lifestyle should be a topic in pre-retirement programs, including health promotion interventions encouraging physical activity, smoking cessation, decreased alcohol intake and a favourable sleep duration. However, it is important to recognise that lifestyle is more than a result of individual choices and lifestyle changes often occur through interaction with others. As a consequence, it is paramount to also look at structural enabling and constraining conditions.

## Supplementary information

**Additional file 1.** Associations between lifestyle risk behaviours and self-rated health, life satisfaction, anxiety and depression.

**Additional file 2.** Question texts, answer categories and coding of independent variables in HUNT2 (1995–97).

**Additional file 3 **12 most prevalent combinations of lifestyle risk behaviours in HUNT2 (1995–97) disregarding missing. *n* = 9101.

**Additional file 4.** Odds ratios (OR) and population attributable fractions (PAF) from adjusted logistic regression analyses.* HUNT2 (1995–97, baseline) and HUNT3 (2006–08, outcome).

**Additional file 5.** Unadjusted logistic regression analyses. A longitudinal study from HUNT2 (1995–97, baseline) to HUNT3 (2006–08, outcome).

**Additional file 6.** Combined effects of lifestyle risk behaviours (HUNT2) on health outcomes (HUNT3), adjusted logistic regression analyses.*

**Additional file 7.** Lifestyle risk behaviours (HUNT2, 1995–97) and odds ratios (OR) for competing outcomes (HUNT3), multinomial logistic regression analyses.*

**Additional file 8.** Lifestyle risk behaviours (HUNT2, 1995–97) and odds ratios (OR) for competing outcomes (HUNT3), multinomial logistic regression analyses.*

**Additional file 9.** Lifestyle risk behaviours (HUNT2, 1995–97) and odds ratios (OR) for competing outcomes (HUNT3), multinomial logistic regression analyses.*

**Additional file 10.** Lifestyle risk behaviours (HUNT2, 1995–97) and odds ratios (OR) for competing outcomes (HUNT3), multinomial logistic regression analyses.*

## Data Availability

The Nord-Trøndelag Health Study (HUNT) has invited persons aged 13–100 years to three surveys between 1994 and 2008, and a fourth survey being completed in 2019 (HUNT4). Comprehensive data from more than 125,000 persons having participated at least once and biological material from78,000 persons are collected. The data are stored in HUNT databank and biological material in HUNT biobank. HUNT Research Centre has been given concession to store and handle these data by the Norwegian Data Inspectorate. The key identification in the data base is the personal identification number given to all Norwegians at birth or immigration, whilst de-identified data are sent to researchers. Due to confidentiality HUNT Research Centre wants to limit storage of data outside HUNT databank, and we have restrictions for researchers for handling of HUNT data files. We have precise information on all data exported to different projects and there are no restrictions regarding data export given approval of applications to HUNT Research Centre. http://www.ntnu.edu/hunt/data

## Abbreviations

CAGE: Questionnaire for risky alcohol consumption; Cut down, Annoyed, Guilty and Eye opener.
HADS: Hospital Anxiety and Depression Scale
HUNT: The Nord-Trøndelag Health Study
OR: Odds Ratio
PAF: Population Attributable Fraction
