# Supplementary material for: Prevalence, clustering and combined effects of lifestyle behaviours and their association with health after retirement age in a prospective cohort study, the Nord-Trøndelag Health Study, Norway
Source: BMC Public Health. 2020 Jun 10;20:900. doi: 10.1186/s12889-020-08993-y (PMC7288686; doi:10.1186/s12889-020-08993-y)
Supplement: Supplementary file 1 — Additional file 1. Associations between lifestyle risk behaviours and self-rated health, life satisfaction, anxiety and depression. [file 12889_2020_8993_MOESM1_ESM.docx]

**Additional file 1.** Associations between lifestyle risk behaviours and self-rated health, life satisfaction, anxiety and depression.

|  | **Self-rated health** | **Life Satisfaction** | **Anxiety** | **Depression** |
| --- | --- | --- | --- | --- |
| **Physical activity** | **Single studies (1-9)** – physical activity is related to self-rated health | **Systematic review and large population-based study (200.000 participants) (9-11)** – positive association between physical activity and quality of life | **Meta-analysis and systematic review  (12-14) –** physical activity associated with lower levels of anxiety | **Meta-analysis, systematic review and review  (12, 15, 16) –** physical activity reduces / protects against depression |
| **Smoking** | **Single studies (17-23)** – smoking associated with poor self-rated health | **Review and large cross-sectional study (n>20.000) (24, 25)** – negative association between smoking and health-related quality of life and quality of life | **Systematic review and meta-analyses (26-28)** – smoking cessation reduce anxiety, but no causal relationship established | **Meta-analyses and systematic review (26-29) –** smoking associated with increased risk of depression, but no causal relationship established |
| **Sitting time** | **Large population study (n>200.000)** **(9)** – sitting time related to self-rated health  **Cross-sectional study (n=3644) (30)** – further research needed | **Systematic review & meta-analysis (mostly cross-sectional studies) and large population study (n>200.000) (9, 31)** – association between sedentary behaviors and physical quality of life and quality of life | **Systematic review (32) –** limited evidence, but a possible association between sitting time and anxiety  **Cross-sectional study (33)** (n=42.469) **–** association between sedentary behaviour and anxiety | **Review (34)** – sedentary behaviour associated with depression but methodological weaknesses  **Meta-analysis (35)** – observational studies indicate association |
| **Sleep disturbance** | **Systematic review and large cross-sectional study (n=63.408) (36, 37)** – short or long sleep duration associated with poor self-rated health | **Meta-analysis (38)** – insomnia negatively associated with quality of life | **Systematic reviews (39, 40) –** bidirectional relationship, causal role unclear | **Meta-analyses & systematic review (41-44)** – insomnia (including short or long sleep duration) increases risk for depression  **Systematic review (39) –** bidirectional relationship |
| **Social participation** | **Large cross-sectional study (n=59.202) (45) –** social participation associated with self-rated health | **Small cross-sectional studies  (46, 47)** –social participation improves quality of life | **Review of Systematic Reviews (48)** – social isolation and loneliness associated with poor mental health  **Systematic review (49)** – social support associated with anxiety | **Systematic reviews** **(48-52)** – heterogeneity between studies |
| **Risky alcohol consumption** | **Single studies (53-61)** – wine and moderate drinking associated with better self-rated health, non-drinkers with worse  In Spain higher alcohol intake was found to be associated with better self-rated health (62) | **Systematic review** **(63)** – alcohol dependence associated with health-related quality of life | **(Critical) Reviews  (64-67)** – complex relationship between alcohol dependence and anxiety | **Review (68)** – causality suggested between alcohol use disorders and major depression  **Meta-analysis (69) –** association between depression and concurrent alcohol usage disorder, depression also associated with future alcohol use and impairment |

**Search strategy in PubMed:** [lifestyle behaviour] AND [outcome]. Filtered first on meta-analyses and systematic reviews. Thereafter large studies. Included if adult populations, preferably if age around retirement age, focusing on underlying associations between lifestyle behaviour and outcome (not interventions). Reference lists in relevant articles were screened.

1. Marques A, Peralta M, Martins J, Catunda R, Matos MG, Saboga Nunes L. Associations between physical activity and self-rated wellbeing in European adults: A population-based, cross-sectional study. Prev Med. 2016;91:18-23.

2. Engberg E, Liira H, Kukkonen-Harjula K, From S, Kautiainen H, Pitkala K, et al. Associations of physical activity with self-rated health and well-being in middle-aged Finnish men. Scand J Public Health. 2015;43(2):190-6.

3. Sodergren M, Sundquist J, Johansson SE, Sundquist K. Physical activity, exercise and self-rated health: a population-based study from Sweden. BMC Public Health. 2008;8:352.

4. Galan I, Meseguer CM, Herruzo R, Rodriguez-Artalejo F. Self-rated health according to amount, intensity and duration of leisure time physical activity. Prev Med. 2010;51(5):378-83.

5. Bodde AE, Seo DC, Frey G. Correlation between physical activity and self-rated health status of non-elderly adults with disabilities. Prev Med. 2009;49(6):511-4.

6. Abu-Omar K, Rutten A, Robine JM. Self-rated health and physical activity in the European Union. Soz Praventivmed. 2004;49(4):235-42.

7. Eriksen L, Curtis T, Gronbaek M, Helge JW, Tolstrup JS. The association between physical activity, cardiorespiratory fitness and self-rated health. Prev Med. 2013;57(6):900-2.

8. Hansen AW, Beyer N, Flensborg-Madsen T, Gronbaek M, Helge JW. Muscle strength and physical activity are associated with self-rated health in an adult Danish population. Prev Med. 2013;57(6):792-8.

9. Rosenkranz RR, Duncan MJ, Rosenkranz SK, Kolt GS. Active lifestyles related to excellent self-rated health and quality of life: cross sectional findings from 194,545 participants in The 45 and Up Study. BMC Public Health. 2013;13:1071.

10. Bize R, Johnson JA, Plotnikoff RC. Physical activity level and health-related quality of life in the general adult population: a systematic review. Prev Med. 2007;45(6):401-15.

11. Pucci GC, Rech CR, Fermino RC, Reis RS. Association between physical activity and quality of life in adults. Rev Saude Publica. 2012;46(1):166-79.

12. Rebar AL, Stanton R, Geard D, Short C, Duncan MJ, Vandelanotte C. A meta-meta-analysis of the effect of physical activity on depression and anxiety in non-clinical adult populations. Health Psychol Rev. 2015;9(3):366-78.

13. Mochcovitch MD, Deslandes AC, Freire RC, Garcia RF, Nardi AE. The effects of regular physical activity on anxiety symptoms in healthy older adults: a systematic review. Braz J Psychiatry. 2016;38(3):255-61.

14. Stubbs B, Koyanagi A, Hallgren M, Firth J, Richards J, Schuch F, et al. Physical activity and anxiety: A perspective from the World Health Survey. J Affect Disord. 2017;208:545-52.

15. Mammen G, Faulkner G. Physical activity and the prevention of depression: a systematic review of prospective studies. Am J Prev Med. 2013;45(5):649-57.

16. Teychenne M, Ball K, Salmon J. Physical activity and likelihood of depression in adults: a review. Prev Med. 2008;46(5):397-411.

17. Svedberg P, Bardage C, Sandin S, Pedersen NL. A prospective study of health, life-style and psychosocial predictors of self-rated health. Eur J Epidemiol. 2006;21(10):767-76.

18. Nakata A, Takahashi M, Swanson NG, Ikeda T, Hojou M. Active cigarette smoking, secondhand smoke exposure at work and home, and self-rated health. Public Health. 2009;123(10):650-6.

19. Manderbacka K, Lundberg O, Martikainen P. Do risk factors and health behaviours contribute to self-ratings of health? Soc Sci Med. 1999;48(12):1713-20.

20. Johansson SE, Sundquist J. Change in lifestyle factors and their influence on health status and all-cause mortality. Int J Epidemiol. 1999;28(6):1073-80.

21. Mood C. Life-style and self-rated global health in Sweden: a prospective analysis spanning three decades. Prev Med. 2013;57(6):802-6.

22. Haveman-Nies A, De Groot LC, Van Staveren WA, Survey in Europe on N, the Elderly: a Concerted Action S. Relation of dietary quality, physical activity, and smoking habits to 10-year changes in health status in older Europeans in the SENECA study. Am J Public Health. 2003;93(2):318-23.

23. Ostbye T, Taylor DH, Jung SH. A longitudinal study of the effects of tobacco smoking and other modifiable risk factors on ill health in middle-aged and old Americans: results from the Health and Retirement Study and Asset and Health Dynamics among the Oldest Old survey. Prev Med. 2002;34(3):334-45.

24. Goldenberg M, Danovitch I, IsHak WW. Quality of life and smoking. Am J Addict. 2014;23(6):540-62.

25. Lopez-Nicolas A, Trapero-Bertran M, Munoz C. Smoking, health-related quality of life and economic evaluation. Eur J Health Econ. 2018;19(5):747-56.

26. Taylor AE, Fluharty ME, Bjorngaard JH, Gabrielsen ME, Skorpen F, Marioni RE, et al. Investigating the possible causal association of smoking with depression and anxiety using Mendelian randomisation meta-analysis: the CARTA consortium. BMJ Open. 2014;4(10):e006141.

27. Taylor G, McNeill A, Girling A, Farley A, Lindson-Hawley N, Aveyard P. Change in mental health after smoking cessation: systematic review and meta-analysis. BMJ. 2014;348:g1151.

28. Baiardini I, Sorino C, F DIM, Facchini F. Smoking cessation, anxiety, mood and quality of life: reassuring evidences. Minerva Med. 2014;105(5 Suppl 1):15-21.

29. Luger TM, Suls J, Vander Weg MW. How robust is the association between smoking and depression in adults? A meta-analysis using linear mixed-effects models. Addict Behav. 2014;39(10):1418-29.

30. Sodergren M, McNaughton SA, Salmon J, Ball K, Crawford DA. Associations between fruit and vegetable intake, leisure-time physical activity, sitting time and self-rated health among older adults: cross-sectional data from the WELL study. BMC Public Health. 2012;12:551.

31. Boberska M, Szczuka Z, Kruk M, Knoll N, Keller J, Hohl DH, et al. Sedentary behaviours and health-related quality of life. A systematic review and meta-analysis. Health Psychol Rev. 2018;12(2):195-210.

32. Teychenne M, Costigan SA, Parker K. The association between sedentary behaviour and risk of anxiety: a systematic review. BMC Public Health. 2015;15:513.

33. Vancampfort D, Stubbs B, Herring MP, Hallgren M, Koyanagi A. Sedentary behavior and anxiety: Association and influential factors among 42,469 community-dwelling adults in six low- and middle-income countries. Gen Hosp Psychiatry. 2017;50:26-32.

34. Teychenne M, Ball K, Salmon J. Sedentary behavior and depression among adults: a review. Int J Behav Med. 2010;17(4):246-54.

35. Zhai L, Zhang Y, Zhang D. Sedentary behaviour and the risk of depression: a meta-analysis. Br J Sports Med. 2015;49(11):705-9.

36. Frange C, de Queiroz SS, da Silva Prado JM, Tufik S, de Mello MT. The impact of sleep duration on self-rated health. Sleep Sci. 2014;7(2):107-13.

37. Magee CA, Caputi P, Iverson DC. Relationships between self-rated health, quality of life and sleep duration in middle aged and elderly Australians. Sleep Med. 2011;12(4):346-50.

38. Ishak WW, Bagot K, Thomas S, Magakian N, Bedwani D, Larson D, et al. Quality of life in patients suffering from insomnia. Innov Clin Neurosci. 2012;9(10):13-26.

39. Alvaro PK, Roberts RM, Harris JK. A Systematic Review Assessing Bidirectionality between Sleep Disturbances, Anxiety, and Depression. Sleep. 2013;36(7):1059-68.

40. Cox RC, Olatunji BO. A systematic review of sleep disturbance in anxiety and related disorders. J Anxiety Disord. 2016;37:104-29.

41. Baglioni C, Battagliese G, Feige B, Spiegelhalder K, Nissen C, Voderholzer U, et al. Insomnia as a predictor of depression: a meta-analytic evaluation of longitudinal epidemiological studies. J Affect Disord. 2011;135(1-3):10-9.

42. Zhai L, Zhang H, Zhang D. Sleep Duration and Depression among Adults: A Meta-Analysis of Prospective Studies. Depress Anxiety. 2015;32(9):664-70.

43. Li L, Wu C, Gan Y, Qu X, Lu Z. Insomnia and the risk of depression: a meta-analysis of prospective cohort studies. BMC Psychiatry. 2016;16(1):375.

44. Bao YP, Han Y, Ma J, Wang RJ, Shi L, Wang TY, et al. Cooccurrence and bidirectional prediction of sleep disturbances and depression in older adults: Meta-analysis and systematic review. Neurosci Biobehav Rev. 2017;75:257-73.

45. Lee HY, Jang SN, Lee S, Cho SI, Park EO. The relationship between social participation and self-rated health by sex and age: a cross-sectional survey. Int J Nurs Stud. 2008;45(7):1042-54.

46. Choi Y, Lee KS, Shin J, Kwon JA, Park EC. Effects of a change in social activity on quality of life among middle-aged and elderly Koreans: Analysis of the Korean longitudinal study of aging (2006-2012). Geriatr Gerontol Int. 2017;17(1):132-41.

47. Bahramnezhad F, Chalik R, Bastani F, Taherpour M, Navab E. The social network among the elderly and its relationship with quality of life. Electron Physician. 2017;9(5):4306-11.

48. Leigh-Hunt N, Bagguley D, Bash K, Turner V, Turnbull S, Valtorta N, et al. An overview of systematic reviews on the public health consequences of social isolation and loneliness. Public Health. 2017;152:157-71.

49. Wang J, Mann F, Lloyd-Evans B, Ma R, Johnson S. Associations between loneliness and perceived social support and outcomes of mental health problems: a systematic review. BMC Psychiatry. 2018;18(1):156.

50. Santini ZI, Koyanagi A, Tyrovolas S, Mason C, Haro JM. The association between social relationships and depression: a systematic review. J Affect Disord. 2015;175:53-65.

51. Schwarzbach M, Luppa M, Forstmeier S, Konig HH, Riedel-Heller SG. Social relations and depression in late life-a systematic review. Int J Geriatr Psychiatry. 2014;29(1):1-21.

52. Gariepy G, Honkaniemi H, Quesnel-Vallee A. Social support and protection from depression: systematic review of current findings in Western countries. Br J Psychiatry. 2016;209(4):284-93.

53. Valencia-Martin JL, Galan I, Rodriguez-Artalejo F. Alcohol and self-rated health in a Mediterranean country: the role of average volume, drinking pattern, and alcohol dependence. Alcohol Clin Exp Res. 2009;33(2):240-6.

54. Poikolainen K, Vartiainen E, Korhonen HJ. Alcohol intake and subjective health. Am J Epidemiol. 1996;144(4):346-50.

55. Poikolainen K, Vartiainen E. Wine and good subjective health. Am J Epidemiol. 1999;150(1):47-50.

56. Theobald H, Johansson SE, Engfeldt P. Influence of different types of alcoholic beverages on self-reported health status. Alcohol Alcohol. 2003;38(6):583-8.

57. Stranges S, Notaro J, Freudenheim JL, Calogero RM, Muti P, Farinaro E, et al. Alcohol drinking pattern and subjective health in a population-based study. Addiction. 2006;101(9):1265-76.

58. Frisher M, Mendonca M, Shelton N, Pikhart H, de Oliveira C, Holdsworth C. Is alcohol consumption in older adults associated with poor self-rated health? Cross-sectional and longitudinal analyses from the English Longitudinal Study of Ageing. BMC Public Health. 2015;15:703.

59. Gronbaek M, Mortensen EL, Mygind K, Andersen AT, Becker U, Gluud C, et al. Beer, wine, spirits and subjective health. J Epidemiol Community Health. 1999;53(11):721-4.

60. Balsa AI, Homer JF, Fleming MF, French MT. Alcohol consumption and health among elders. Gerontologist. 2008;48(5):622-36.

61. Moriconi PA, Nadeau L. A Cross-Sectional Study of Self-Rated Health among Older Adults: Association with Drinking Profiles and Other Determinants of Health. Curr Gerontol Geriatr Res. 2015;2015:352947.

62. Guallar-Castillon P, Rodriguez-Artalejo F, Diez Ganan LD, Banegas Banegas JR, Lafuente Urdinguio PL, Herruzo Cabrera RH. Consumption of alcoholic beverages and subjective health in Spain. J Epidemiol Community Health. 2001;55(9):648-52.

63. Levola J, Aalto M, Holopainen A, Cieza A, Pitkanen T. Health-related quality of life in alcohol dependence: a systematic literature review with a specific focus on the role of depression and other psychopathology. Nord J Psychiatry. 2014;68(6):369-84.

64. Morris EP, Stewart SH, Ham LS. The relationship between social anxiety disorder and alcohol use disorders: a critical review. Clin Psychol Rev. 2005;25(6):734-60.

65. Kushner MG, Abrams K, Borchardt C. The relationship between anxiety disorders and alcohol use disorders: A review of major perspectives and findings. Clinical Psychology Review. 2000;20(2):149-71.

66. Schuckit MA, Hesselbrock V. Alcohol dependence and anxiety disorders: what is the relationship? Am J Psychiatry. 1994;151(12):1723-34.

67. Allan CA. Alcohol problems and anxiety disorders--a critical review. Alcohol Alcohol. 1995;30(2):145-51.

68. Boden JM, Fergusson DM. Alcohol and depression. Addiction. 2011;106(5):906-14.

69. Conner KR, Pinquart M, Gamble SA. Meta-analysis of depression and substance use among individuals with alcohol use disorders. J Subst Abuse Treat. 2009;37(2):127-37.
