# Supplementary material for: Prevalence, clustering and combined effects of lifestyle behaviours and their association with health after retirement age in a prospective cohort study, the Nord-Trøndelag Health Study, Norway
Source: BMC Public Health. 2020 Jun 10;20:900. doi: 10.1186/s12889-020-08993-y (PMC7288686; doi:10.1186/s12889-020-08993-y)
Supplement: Supplementary file 2 — Additional file 2. Question texts, answer categories and coding of independent variables in HUNT2 (1995–97). [file 12889_2020_8993_MOESM2_ESM.docx]

**Additional file 2.** Question texts, answer categories and coding of independent variables in HUNT2 (1995-97).

| **Variable HUNT2** | **Question text (from HUNT)** | **Answer categories (from HUNT)** | **Variable coding** |
| --- | --- | --- | --- |
| **Smoking** | Do you smoke?  Corrected for answers in HUNT1 and HUNT3 | Never smoked daily  Ex-smoker daily  Current smoker daily | **Current smoker daily**  **Not current smoker daily** |
| **Alcohol** | Have you ever felt that you should reduce your alcohol intake?  Have other people ever criticised your use of alcohol?  Have you ever felt bad or guilty because of your use of alcohol?  Have you ever had a drink first thing in the morning as a pick-me-up or to calm your nerves or to cure a hangover? | No = 0  Yes = 1 | **Risky alcohol consumption** = ≥ 2 yes  **Not risky alcohol consumption** = ≤ 1 yes |
| **Sleep duration** | How many hours do you usually spend lying down during a 24-hour period? | Report number of hours | **Short or prolonged sleeping time** = ≤ 6 or ≥ 10 hours  **Normal sleeping hours** = 7-9 hours |
| **Sitting time** | How many hours do you usually spend sitting down during a 24-hour period? | Report number of hours | **Prolonged sitting time** = ≥ 8 hours  **Normal sitting time** = ≤ 7 hours |
| **Social participation** | How often do you usually participate in social activities such as a sewing club, athletic club, political association, religious or other groups? | Never, or only a few times a year  1-2 times a month  About once a week  More than once a week | **Never or seldom socially active** = never, or only a few times a year  **Socially active** = 1-2 times a month + about once a week + more than once a week |
| **Physical activity** | Average of hours of low physical activity per week in the last year (not panting for breath or sweating)?  Average hours of vigorous physical activity per week in the last year (panting for breath or sweating)? | None  Less than 1 hour  1-2 hours  3 hours or more | **Physically inactive** = ≤ 3 hours light & no hard physical activity per week  **Physically active** = 3 hours or more light physical activity or ≥ 1 hour hard physical activity |
