# Supplementary material for: Prevalence, clustering and combined effects of lifestyle behaviours and their association with health after retirement age in a prospective cohort study, the Nord-Trøndelag Health Study, Norway
Source: BMC Public Health. 2020 Jun 10;20:900. doi: 10.1186/s12889-020-08993-y (PMC7288686; doi:10.1186/s12889-020-08993-y)
Supplement: Supplementary file 3 — Additional file 3 12 most prevalent combinations of lifestyle risk behaviours in HUNT2 (1995–97) disregarding missing. n = 9101. [file 12889_2020_8993_MOESM3_ESM.docx]

| **Additional file 3.** 12 most prevalent combinations of lifestyle risk behaviours in HUNT2 (1995-97) disregarding missing. n=9101 | | | | | | | | |
| --- | --- | --- | --- | --- | --- | --- | --- | --- |
| Combination | Prevalence (%) | Cumulative % | Smoking | Alcohol | Sitting | Inactive | Social | Sleep |
| 1 | 1618 (17.8) | 17.8 | - | - | - | - | - | - |
| 2 | 1060 (11.7) | 29.4 | - | - | - | + | - | - |
| 3 | 883 (9.7) | 39.1 | - | - | - | - | + | - |
| 4 | 617 (6.8) | 45.9 | - | - | - | + | + | - |
| 5 | 569 (6.3) | 52.2 | + | - | - | - | - | - |
| 6 | 515 (5.7) | 57.8 | - | - | + | - | - | - |
| 7 | 390 (4.3) | 62.1 | + | - | - | - | + | - |
| 8 | 373 (4.1) | 66.2 | + | - | - | + | - | - |
| 9 | 339 (3.7) | 69.9 | + | - | - | + | + | - |
| 10 | 306 (3.4) | 73.3 | - | - | + | - | + | - |
| 11 | 279 (3.1) | 76.4 | - | - | + | + | - | - |
| 12 | 240 (2.7) | 79.0 | - | - | + | + | + | - |
| Total | 7189 (79.0) |  |  |  |  |  |  |  |
