# Supplementary material for: Prevalence, clustering and combined effects of lifestyle behaviours and their association with health after retirement age in a prospective cohort study, the Nord-Trøndelag Health Study, Norway
Source: BMC Public Health. 2020 Jun 10;20:900. doi: 10.1186/s12889-020-08993-y (PMC7288686; doi:10.1186/s12889-020-08993-y)
Supplement: Supplementary file 4 — Additional file 4. Odds ratios (OR) and population attributable fractions (PAF) from adjusted logistic regression analyses.* HUNT2 (1995–97, baseline) and HUNT3 (2006–08, outcome). [file 12889_2020_8993_MOESM4_ESM.docx]

| **Additional file 4.** Odds ratios (OR) and population attributable fractions (PAF) from adjusted logistic regression analyses.* HUNT2 (1995-97, baseline) and HUNT3 (2006-08, outcome). | | | | | | | | | | | | | | | | | | | | | | |
| --- | --- | --- | --- | --- | --- | --- | --- | --- | --- | --- | --- | --- | --- | --- | --- | --- | --- | --- | --- | --- | --- | --- |
|  |  |  |  | Self-rated health | | | |  | Life satisfaction | | | |  | Anxiety | | | |  | Depression | | | |
|  |  |  |  | Good | Poor | | |  | Good | Poor | | |  | No | Yes | | |  | No | Yes | | |
|  |  |  |  | n | n |  | 95% CI |  | n | n |  | 95% CI |  | n | n |  | 95% CI |  | n | n |  | 95% CI |
| Daily smoker | | |  |  |  |  |  |  |  |  |  |  |  |  |  |  |  |  |  |  |  |  |
|  | No | OR |  | 2349 | 546 | 1.00 | ref |  | 3566 | 203 | 1.00 | ref |  | 2756 | 171 | 1.00 | ref |  | 3081 | 219 | 1.00 | ref |
|  | Yes |  |  | 599 | 204 | 1.40 | (1.16-1.69) |  | 966 | 65 | 1.13 | (0.84-1.52) |  | 749 | 52 | 1.06 | (0.76-1.47) |  | 863 | 76 | 1.19 | (0.90-1.57) |
|  |  | PAF |  |  |  | 6.17 | (2.51-9.69) |  |  |  | 2.62 | (-4.03-8.85) |  |  |  | 1.20 | (-6.00-7.92) |  |  |  | 3.83 | (-2.65-9.89) |
|  |  | Total |  | 3698 |  |  |  |  | 4800 |  |  |  |  | 3728 |  |  |  |  | 4239 |  |  |  |
| Physical activity | | |  |  |  |  |  |  |  |  |  |  |  |  |  |  |  |  |  |  |  |  |
|  | Active | OR |  | 1720 | 374 | 1.00 | ref |  | 2428 | 130 | 1.00 | ref |  | 1930 | 119 | 1.00 | ref |  | 2165 | 131 | 1.00 | ref |
|  | Inactive |  |  | 1042 | 320 | 1.31 | (1.11-1.56) |  | 1800 | 116 | 1.12 | (0.86-1.46) |  | 1425 | 96 | 0.88 | (0.66-1.16) |  | 1554 | 143 | 1.44 | (1.12-1.85) |
|  |  | PAF |  |  |  | 8.79 | (3.02-14.2) |  |  |  | 4.65 | (-7.12-15.1) |  |  |  | -5.80 | (-18.95-5.89) |  |  |  | 14.92 | (4.02-24.58) |
|  |  | Total |  | 3456 |  |  |  |  | 4474 |  |  |  |  | 3570 |  |  |  |  | 3993 |  |  |  |
| Sitting time | | |  |  |  |  |  |  |  |  |  |  |  |  |  |  |  |  |  |  |  |  |
|  | ≤ 7 hours | OR |  | 1639 | 427 | 1.00 | ref |  | 2599 | 160 | 1.00 | ref |  | 1989 | 138 | 1.00 | ref |  | 2292 | 177 | 1.00 | ref |
|  | ≥ 8 hours |  |  | 840 | 197 | 0.98 | (0.81-1.19) |  | 1221 | 71 | 0.96 | (0.72-1.29) |  | 1091 | 61 | 1.00 | (0.73-1.38) |  | 1141 | 82 | 1.01 | (0.77-1.34) |
|  |  | PAF |  |  |  | -0.47 | (-5.54-4.35) |  |  |  | -1.10 | (-10.09-7.15) |  |  |  | -0.02 | (-9.54-8.67) |  |  |  | 0.34 | (-8.14-8.15) |
|  |  | Total |  | 3103 |  |  |  |  | 4051 |  |  |  |  | 3279 |  |  |  |  | 3692 |  |  |  |
| Alcohol | | |  |  |  |  |  |  |  |  |  |  |  |  |  |  |  |  |  |  |  |  |
|  | CAGE ≤ 1 | OR |  | 2131 | 516 | 1.00 | ref |  | 3198 | 176 | 1.00 | ref |  | 2585 | 165 | 1.00 | ref |  | 2882 | 206 | 1.00 | ref |
|  | CAGE ≥ 2 |  |  | 129 | 41 | 1.41 | (0.97-2.06) |  | 190 | 14 | 1.47 | (0.82-2.62) |  | 157 | 12 | 1.92 | (1.02-3.62) |  | 167 | 18 | 1.61 | (0.96-2.72) |
|  |  | PAF |  |  |  | 1.72 | (-0.32-3.71) |  |  |  | 2.21 | (-1.68-5.96) |  |  |  | 3.09 | (-0.71-6.73) |  |  |  | 2.84 | (-0.83-6.37) |
|  |  | Total |  | 2817 |  |  |  |  | 3578 |  |  |  |  | 2919 |  |  |  |  | 3273 |  |  |  |
| Social participation | | |  |  |  |  |  |  |  |  |  |  |  |  |  |  |  |  |  |  |  |  |
|  | Participates | OR |  | 1518 | 363 | 1.00 | ref |  | 2250 | 135 | 1.00 | ref |  | 1801 | 112 | 1.00 | ref |  | 2028 | 137 | 1.00 | ref |
|  | Seldom, never | |  | 1132 | 302 | 1.07 | (0.90-1.28) |  | 1824 | 104 | 0.97 | (0.74-1.28) |  | 1439 | 100 | 1.14 | (0.85-1.52) |  | 1602 | 136 | 1.18 | (0.92-1.53) |
|  |  | PAF |  |  |  | 2.44 | (-3.89-8.38) |  |  |  | -1.07 | (-13.0-9.58) |  |  |  | 5.35 | (-7.28-16.49) |  |  |  | 7.15 | (-4.32-17.36) |
|  |  | Total |  | 3315 |  |  |  |  | 4313 |  |  |  |  | 3452 |  |  |  |  | 3903 |  |  |  |
| Sleep | | |  |  |  |  |  |  |  |  |  |  |  |  |  |  |  |  |  |  |  |  |
|  | 7-9 hours | OR |  | 2369 | 569 | 1.00 | ref |  | 3542 | 196 | 1.00 | ref |  | 2837 | 172 | 1.00 | ref |  | 3171 | 231 | 1.00 | ref |
|  | ≤ 6 / ≥ 10 hours | |  | 248 | 94 | 1.49 | (1.15-1.93) |  | 498 | 44 | 1.47 | (1.04-2.08) |  | 376 | 37 | 1.39 | (0.95-2.04) |  | 428 | 42 | 1.23 | (0.87-1.74) |
|  |  | PAF |  |  |  | 3.69 | (1.11-6.20) | |  |  | 5.53 | (-0.08-10.82) | | |  | 4.60 | (-1.29-10.16) | | |  | 2.65 | (-2.19-7.26) |
|  |  | Total |  | 3280 |  |  |  | | 4280 |  |  |  | | | 3422 |  |  | | 3872 |  | |  |
| *Adjusted for age, sex, education, marital status and chronic illness  n varies from 2817 to 4800 due to different amount of missing on the variables  Abbreviations used in the table: CAGE = screening questionnaire for risky alcohol consumption, HUNT = the Nord-Trøndelag Health Study, OR = Odds Ratio, PAF = Population Attributable Fraction in %, ref = reference category | | | | | | | | | | | | | | | | | | | | | | |
