# Supplementary material for: Prevalence, clustering and combined effects of lifestyle behaviours and their association with health after retirement age in a prospective cohort study, the Nord-Trøndelag Health Study, Norway
Source: BMC Public Health. 2020 Jun 10;20:900. doi: 10.1186/s12889-020-08993-y (PMC7288686; doi:10.1186/s12889-020-08993-y)
Supplement: Supplementary file 5 — Additional file 5. Unadjusted logistic regression analyses. A longitudinal study from HUNT2 (1995–97, baseline) to HUNT3 (2006–08, outcome). [file 12889_2020_8993_MOESM5_ESM.docx]

| **Additional file 5.** Unadjusted logistic regression analyses. A longitudinal study from HUNT2 (1995-97, baseline) to HUNT3 (2006-08, outcome). | | | | | | | | | | | | | | | | | | | | | | |
| --- | --- | --- | --- | --- | --- | --- | --- | --- | --- | --- | --- | --- | --- | --- | --- | --- | --- | --- | --- | --- | --- | --- |
|  |  |  | | Self-rated health | | | |  | Life satisfaction | | | |  | Anxiety | | | |  | Depression | | | |
|  |  |  | | Good | Poor | | |  | Good | Poor | | |  | No | Yes | | |  | No | Yes | | |
|  |  |  | | n | n | OR | 95% CI |  | n | n | OR | 95% CI |  | n | n | OR | 95% CI |  | n | n | OR | 95% CI |
| Daily smoking | | | | |  |  |  |  |  |  |  |  |  |  |  |  |  |  |  |  |  |  |
|  | no | | 2423 | | 574 | 1.00 | ref |  | 3715 | 212 | 1.00 | ref |  | 2847 | 173 | 1.00 | ref |  | 3194 | 228 | 1.00 | ref |
|  | yes | | 618 | | 212 | 1.45 | (1.21-1.73) |  | 1006 | 70 | 1.22 | (0.92-1.61) |  | 770 | 54 | 1.15 | (0.84-1.58) |  | 894 | 81 | 1.27 | (0.97-1.65) |
|  | total |  | | 3827 |  |  |  |  | 5003 |  |  |  |  | 3844 |  |  |  |  | 4397 |  |  |  |
| Physical activity | | | | |  |  |  |  |  |  |  |  |  |  |  |  |  |  |  |  |  |  |
|  | active | | 1757 | | 386 | 1.00 | ref |  | 2495 | 133 | 1.00 | ref |  | 1976 | 119 | 1.00 | ref |  | 2224 | 134 | 1.00 | ref |
|  | inactive | | 1080 | | 336 | 1.42 | (1.20-1.67) |  | 1885 | 125 | 1.24 | (0.97-1.60) |  | 1476 | 100 | 1.13 | (0.86-1.48) |  | 1620 | 150 | 1.54 | (1.21-1.96) |
|  | total |  | | 3559 |  |  |  |  | 4638 |  |  |  |  | 3671 |  |  |  |  | 4128 |  |  |  |
| Sitting time | | | | |  |  |  |  |  |  |  |  |  |  |  |  |  |  |  |  |  |  |
|  | ≤ 7 hours | | 1688 | | 451 | 1.00 | ref |  | 2701 | 171 | 1.00 | ref |  | 2053 | 140 | 1.00 | ref |  | 2369 | 186 | 1.00 | ref |
|  | ≥ 8 hours | | 865 | | 203 | 0.88 | (0.73-1.06) |  | 1267 | 71 | 0.89 | (0.67-1.18) |  | 1122 | 63 | 0.82 | (0.61-1.12) |  | 1183 | 84 | 0.90 | (0.69-1.18) |
|  | total |  | | 3207 |  |  |  |  | 4210 |  |  |  |  | 3378 |  |  |  |  | 3822 |  |  |  |
| Alcohol | | | | |  |  |  |  |  |  |  |  |  |  |  |  |  |  |  |  |  |  |
|  | CAGE ≤ 1 | | 2194 | | 539 | 1.00 | ref |  | 3323 | 183 | 1.00 | ref |  | 2667 | 168 | 1.00 | ref |  | 2986 | 216 | 1.00 | ref |
|  | CAGE ≥ 2 | | 132 | | 42 | 1.30 | (0.90-1.86) |  | 196 | 14 | 1.30 | (0.74-2.28) |  | 157 | 12 | 1.21 | (0.66-2.23) |  | 169 | 18 | 1.47 | (0.89-2.44) |
|  | total |  | | 2907 |  |  |  |  | 3716 |  |  |  |  | 3004 |  |  |  |  | 3389 |  |  |  |
| Social participation | | | | |  |  |  |  |  |  |  |  |  |  |  |  |  |  |  |  |  |  |
|  | participates | | 1566 | | 376 | 1.00 | ref |  | 2339 | 139 | 1.00 | ref |  | 1854 | 115 | 1.00 | ref |  | 2096 | 145 | 1.00 | ref |
|  | seldom, never | | 1166 | | 322 | 1.15 | (0.97-1.36) |  | 1901 | 113 | 1.00 | (0.77-1.29) |  | 1488 | 101 | 1.09 | (0.83-1.44) |  | 1665 | 141 | 1.22 | (0.96-1.56) |
|  | total |  | | 3430 |  |  |  |  | 4492 |  |  |  |  | 3558 |  |  |  |  | 4047 |  |  |  |
| Sleep duration | | | | |  |  |  |  |  |  |  |  |  |  |  |  |  |  |  |  |  |  |
|  | 7-9 hours | | 2440 | | 595 | 1.00 | ref |  | 3677 | 209 | 1.00 | ref |  | 2924 | 167 | 1.00 | ref |  | 3284 | 239 | 1.00 | ref |
|  | ≤ 6 / ≥ 10 hours | | 260 | | 99 | 1.56 | (1.22-2.00) |  | 523 | 44 | 1.48 | (1.06-2.07) |  | 387 | 37 | 1.59 | (1.10-2.30) |  | 443 | 46 | 1.43 | (1.02-1.99) |
|  | total |  | | 3394 |  |  |  |  | 4453 |  |  |  |  | 3524 |  |  |  |  | 4012 |  |  |  |
| n varies from 2907 to 5003 due to different amount of missing on the variables | | | | | | | | | | | | | | | | | | | | | | |
| Abbreviations used in the table: CAGE = screening questionnaire for risky alcohol consumption, CI = Confidence Interval, HUNT = the Nord-Trøndelag Health Study, OR = Odds Ratio, ref = reference category | | | | | | | | | | | | | | | | | | | | | | |
