# Supplementary material for: Prevalence, clustering and combined effects of lifestyle behaviours and their association with health after retirement age in a prospective cohort study, the Nord-Trøndelag Health Study, Norway
Source: BMC Public Health. 2020 Jun 10;20:900. doi: 10.1186/s12889-020-08993-y (PMC7288686; doi:10.1186/s12889-020-08993-y)
Supplement: Supplementary file 6 — Additional file 6. Combined effects of lifestyle risk behaviours (HUNT2) on health outcomes (HUNT3), adjusted logistic regression analyses.* [file 12889_2020_8993_MOESM6_ESM.docx]

| **Additional file 6.** Combined effects of lifestyle risk behaviours (HUNT2) on health outcomes (HUNT3), adjusted logistic regression analyses.* | | | | | | | | | | | | | | | | | | | | | | | | | |
| --- | --- | --- | --- | --- | --- | --- | --- | --- | --- | --- | --- | --- | --- | --- | --- | --- | --- | --- | --- | --- | --- | --- | --- | --- | --- |
|  |  |  | Self-rated health | | | | |  | Life satisfaction | | | | |  | Anxiety | | | | |  | Depression | | | | |
|  |  |  | Good |  | Poor | | |  | Good |  | Poor | | |  | No |  | Yes | | |  | No |  | Yes | | |
|  |  |  | n |  | n | OR | 95% CI |  | n |  | n | OR | 95% CI |  | n |  | n | OR | 95% CI |  | n |  | n | OR | 95% CI |
| Risk factors | | | |  |  |  |  |  |  |  |  |  |  |  |  |  |  |  |  |  |  |  |  |  |  |
|  | None |  | 660 |  | 149 | 1.00 | Ref |  | 923 |  | 50 | 1.00 | Ref |  | 659 |  | 38 | 1.00 | Ref |  | 774 |  | 48 | 1.00 | Ref |
|  | One |  | 1139 |  | 265 | 0.99 | (0.79-1.24) |  | 1687 |  | 94 | 1.02 | (0.71-1.45) |  | 1288 |  | 80 | 1.04 | (0.69-1.55) |  | 1469 |  | 94 | 0.99 | (0.69-1.42) |
|  | Two |  | 784 |  | 197 | 1.06 | (0.83-1.34) |  | 1270 |  | 79 | 1.09 | (0.75-1.57) |  | 968 |  | 63 | 1.11 | (0.73-1.69) |  | 1090 |  | 90 | 1.26 | (0.88-1.82) |
|  | Three |  | 312 |  | 98 | 1.34 | (1.00-1.79) |  | 541 |  | 36 | 1.18 | (0.76-1.84) |  | 462 |  | 32 | 1.16 | (0.71-1.90) |  | 485 |  | 48 | 1.50 | (0.99-2.28) |
|  | ≥ Four | | 83 |  | 48 | 2.39 | (1.60-3.58) |  | 154 |  | 13 | 1.43 | (0.75-2.72) |  | 151 |  | 13 | 1.48 | (0.76-2.89) |  | 157 |  | 18 | 1.72 | (0.97-3.06) |
| Total | |  | 3735 |  |  |  |  |  | 4847 |  |  |  |  |  | 3754 |  |  |  |  |  | 4273 |  |  |  |  |
| *Adjusted for age, sex, education, marital status and chronic illness  Abbreviations used in the table: CI = Confidence Interval, OR = Odds Ratio, Ref = reference group | | | | | | | | | | | | | | | | | | | | | | | |  |  |
