# Supplementary material for: Prevalence, clustering and combined effects of lifestyle behaviours and their association with health after retirement age in a prospective cohort study, the Nord-Trøndelag Health Study, Norway
Source: BMC Public Health. 2020 Jun 10;20:900. doi: 10.1186/s12889-020-08993-y (PMC7288686; doi:10.1186/s12889-020-08993-y)
Supplement: Supplementary file 7 — Additional file 7. Lifestyle risk behaviours (HUNT2, 1995–97) and odds ratios (OR) for competing outcomes (HUNT3), multinomial logistic regression analyses.* [file 12889_2020_8993_MOESM7_ESM.docx]

| **Additional file 7**. Lifestyle risk behaviours (HUNT2, 1995-97) and odds ratios (OR) for competing outcomes (HUNT3), multinomial logistic regression analyses.* | | | | | | | | | | | | | | |
| --- | --- | --- | --- | --- | --- | --- | --- | --- | --- | --- | --- | --- | --- | --- |
|  |  | Good health |  | Poor health | | |  | Non-participation in HUNT3 | | |  | Mortality during follow-up | | |
|  |  | n |  | n | OR | 95% CI |  | n | OR | 95% CI |  | n | OR | 95% CI |
| Daily smoking | |  |  |  |  |  |  |  |  |  |  |  |  |  |
|  | no | 2349 |  | 546 | 1.00 | ref |  | 606 | 1.00 | ref |  | 211 | 1.00 | ref |
|  | yes | 599 |  | 204 | 1.42 | (1.18-1.72) |  | 413 | 2.50 | (2.13-2.93) |  | 139 | 2.47 | (1.95-3.14) |
|  | total | 5067 |  |  |  |  |  |  |  |  |  |  |  |  |
| Physical activity | |  |  |  |  |  |  |  |  |  |  |  |  |  |
|  | active | 1720 |  | 374 | 1.00 | ref |  | 480 | 1.00 | ref |  | 172 | 1.00 | ref |
|  | inactive | 1042 |  | 320 | 1.32 | (1.11-1.57) |  | 428 | 1.38 | (1.18-1.61) |  | 142 | 1.37 | (1.08-1.74) |
|  | total | 4678 |  |  |  |  |  |  |  |  |  |  |  |  |
| Sitting time | |  |  |  |  |  |  |  |  |  |  |  |  |  |
|  | ≤ 7 hours | 1639 |  | 427 | 1.00 | ref |  | 486 | 1.00 | ref |  | 167 | 1.00 | ref |
|  | ≥ 8 hours | 840 |  | 197 | 0.99 | (0.81-1.20) |  | 223 | 1.02 | (0.84-1.23) |  | 98 | 1.20 | (0.91-1.57) |
|  | total | 4077 |  |  |  |  |  |  |  |  |  |  |  |  |
| Alcohol | |  |  |  |  |  |  |  |  |  |  |  |  |  |
|  | CAGE ≤ 1 | 2131 |  | 516 | 1.00 | ref |  | 696 | 1.00 | ref |  | 224 | 1.00 | ref |
|  | CAGE ≥ 2 | 129 |  | 41 | 1.43 | (0.98-2.08) |  | 44 | 1.25 | (0.86-1.80) |  | 21 | 1.31 | (0.80-2.15) |
|  | total | 3702 |  |  |  |  |  |  |  |  |  |  |  |  |
| Social participation | |  |  |  |  |  |  |  |  |  |  |  |  |  |
|  | participates | 1518 |  | 363 | 1.00 | ref |  | 352 | 1.00 | ref |  | 127 | 1.00 | ref |
|  | seldom, never | 1132 |  | 302 | 1.06 | (0.88-1.26) |  | 434 | 1.41 | (1.19-1.67) |  | 158 | 1.36 | (1.05-1.76) |
|  | total | 4386 |  |  |  |  |  |  |  |  |  |  |  |  |
| Sleep duration | |  |  |  |  |  |  |  |  |  |  |  |  |  |
|  | 7-9 hours | 2369 |  | 569 | 1.00 | ref |  | 683 | 1.00 | ref |  | 243 | 1.00 | ref |
|  | ≤ 6 or ≥ 10 hours | 248 |  | 94 | 1.48 | (1.14.1.92) |  | 98 | 1.22 | (0.94-1.57) |  | 37 | 1.38 | (0.95-2.01) |
|  | total | 4341 |  |  |  |  |  |  |  |  |  |  |  |  |
| *Adjusted for age, sex, education, marital status and chronic illness | | | | | | | | | |  |  |  |  |  |
| n varies from 3702 to 5067 due to different amount of missing on the lifestyle variables | | | | | | | | | | |  |  |  |  |
| Abbreviations used in the table: CAGE = screening questionnaire for risky alcohol consumption, CI = Confidence interval, HUNT = the Nord-Trøndelag Health Study, OR = Odds Ratio | | | | | | | | | | | | | | |
