# Supplementary material for: Prevalence, clustering and combined effects of lifestyle behaviours and their association with health after retirement age in a prospective cohort study, the Nord-Trøndelag Health Study, Norway
Source: BMC Public Health. 2020 Jun 10;20:900. doi: 10.1186/s12889-020-08993-y (PMC7288686; doi:10.1186/s12889-020-08993-y)
Supplement: Supplementary file 8 — Additional file 8. Lifestyle risk behaviours (HUNT2, 1995–97) and odds ratios (OR) for competing outcomes (HUNT3), multinomial logistic regression analyses.* [file 12889_2020_8993_MOESM8_ESM.docx]

| **Additional file 8.** Lifestyle risk behaviours (HUNT2, 1995-97) and odds ratios (OR) for competing outcomes (HUNT3), multinomial logistic regression analyses.* | | | | | | | | | | | | | | |
| --- | --- | --- | --- | --- | --- | --- | --- | --- | --- | --- | --- | --- | --- | --- |
|  |  | Good life satisfaction |  | Poor life satisfaction | | |  | Non-participation in HUNT3 | | |  | Mortality during follow-up | | |
|  |  | n |  | n | OR | 95% CI |  | n | OR | 95% CI |  | n | OR | 95% CI |
| Daily smoking | |  |  |  |  |  |  |  |  |  |  |  |  |  |
|  | no | 3566 |  | 203 | 1.00 | ref |  | 833 | 1.00 | ref |  | 308 | 1.00 | ref |
|  | yes | 966 |  | 65 | 1.15 | (0.86-1.54) |  | 552 | 2.28 | (2.00-2.60) |  | 208 | 2.43 | (2.00-2.95) |
|  | total | 6701 |  |  |  |  |  |  |  |  |  |  |  |  |
| Physical activity | |  |  |  |  |  |  |  |  |  |  |  |  |  |
|  | active | 2428 |  | 130 | 1.00 | ref |  | 635 | 1.00 | ref |  | 228 | 1.00 | ref |
|  | inactive | 1800 |  | 116 | 1.13 | (0.87-1.48) |  | 605 | 1.24 | (1.08-1.41) |  | 226 | 1.38 | (1.13-1.68) |
|  | total | 6168 |  |  |  |  |  |  |  |  |  |  |  |  |
| Sitting time | |  |  |  |  |  |  |  |  |  |  |  |  |  |
|  | ≤ 7 hours | 2599 |  | 160 | 1.00 | ref |  | 644 | 1.00 | ref |  | 249 | 1.00 | ref |
|  | ≥ 8 hours | 1221 |  | 71 | 0.96 | (0.72-1.29) |  | 316 | 1.15 | (0.98-1.34) |  | 145 | 1.28 | (1.03-1.60) |
|  | total | 5405 |  |  |  |  |  |  |  |  |  |  |  |  |
| Alcohol | |  |  |  |  |  |  |  |  |  |  |  |  |  |
|  | CAGE ≤ 1 | 3198 |  | 176 | 1.00 | ref |  | 814 | 1.00 | ref |  | 333 | 1.00 | ref |
|  | CAGE ≥ 2 | 190 |  | 14 | 1.43 | (0.80-2.56) |  | 52 | 1.06 | (0.76-1.47) |  | 30 | 1.32 | (0.87-2.00) |
|  | total | 4807 |  |  |  |  |  |  |  |  |  |  |  |  |
| Social participation | |  |  |  |  |  |  |  |  |  |  |  |  |  |
|  | participates | 2250 |  | 135 | 1.00 | ref |  | 471 | 1.00 | ref |  | 186 | 1.00 | ref |
|  | seldom, never | 1824 |  | 104 | 0.96 | (0.73-1.27) |  | 605 | 1.37 | (1.19-1.58) |  | 243 | 1.32 | (1.07-1.63) |
|  | total | 5818 |  |  |  |  |  |  |  |  |  |  |  |  |
| Sleep duration | |  |  |  |  |  |  |  |  |  |  |  |  |  |
|  | 7-9 hours | 3542 |  | 196 | 1.00 | ref |  | 904 | 1.00 | ref |  | 362 | 1.00 | ref |
|  | ≤ 6 or ≥ 10 hours | 498 |  | 44 | 1.45 | (1.03-2.05) |  | 159 | 1.11 | (0.91-1.35) |  | 66 | 1.21 | (0.91-1.61) |
|  | total | 5771 |  |  |  |  |  |  |  |  |  |  |  |  |
| *Adjusted for age, sex, education, marital status and chronic illness | | | | | | | | | |  |  |  |  |  |
| n varies from 4807 to 6701 due to different amount of missing on the lifestyle variables | | | | | | | | | | |  |  |  |  |
| Abbreviations used in the table: CAGE = screening questionnaire for risky alcohol consumption, CI = Confidence interval, HUNT = the Nord-Trøndelag Health Study, OR = Odds Ratio | | | | | | | | | | | | | | |
