# Supplementary material for: Prevalence, clustering and combined effects of lifestyle behaviours and their association with health after retirement age in a prospective cohort study, the Nord-Trøndelag Health Study, Norway
Source: BMC Public Health. 2020 Jun 10;20:900. doi: 10.1186/s12889-020-08993-y (PMC7288686; doi:10.1186/s12889-020-08993-y)
Supplement: Supplementary file 9 — Additional file 9. Lifestyle risk behaviours (HUNT2, 1995–97) and odds ratios (OR) for competing outcomes (HUNT3), multinomial logistic regression analyses.* [file 12889_2020_8993_MOESM9_ESM.docx]

| **Additional file 9**. Lifestyle risk behaviours (HUNT2, 1995-97) and odds ratios (OR) for competing outcomes (HUNT3), multinomial logistic regression analyses.* | | | | | | | | | | | | | | |
| --- | --- | --- | --- | --- | --- | --- | --- | --- | --- | --- | --- | --- | --- | --- |
|  |  | No anxiety |  | Anxiety | | |  | Non-participation HUNT3 | | |  | Mortality during follow-up | | |
|  |  | n |  | n | OR | 95% CI |  | n | OR | 95% CI |  | n | OR | 95% CI |
| Daily smoking | |  |  |  |  |  |  |  |  |  |  |  |  |  |
|  | no | 2756 |  | 171 | 1.00 | ref |  | 702 | 1.00 | ref |  | 262 | 1.00 | ref |
|  | yes | 749 |  | 52 | 1.07 | (0.78-1.49) |  | 463 | 2.24 | (1.94-2.60) |  | 196 | 2.63 | (2.14-3.24) |
|  | total | 5351 |  |  |  |  |  |  |  |  |  |  |  |  |
| Physical activity | |  |  |  |  |  |  |  |  |  |  |  |  |  |
|  | active | 1930 |  | 119 | 1.00 | ref |  | 557 | 1.00 | ref |  | 212 | 1.00 | ref |
|  | inactive | 1425 |  | 96 | 0.89 | (0.67-1.18) |  | 520 | 1.22 | (1.06-1.41) |  | 203 | 1.35 | (1.09-1.67) |
|  | total | 5062 |  |  |  |  |  |  |  |  |  |  |  |  |
| Sitting time | |  |  |  |  |  |  |  |  |  |  |  |  |  |
|  | ≤ 7 hours | 1989 |  | 138 | 1.00 | ref |  | 550 | 1.00 | ref |  | 219 | 1.00 | ref |
|  | ≥ 8 hours | 1091 |  | 61 | 0.99 | (0.72-1.35) |  | 295 | 1.08 | (0.92-1.28) |  | 152 | 1.33 | (1.06-1.68) |
|  | total | 4495 |  |  |  |  |  |  |  |  |  |  |  |  |
| Alcohol | |  |  |  |  |  |  |  |  |  |  |  |  |  |
|  | CAGE ≤ 1 | 2585 |  | 165 | 1.00 | ref |  | 706 | 1.00 | ref |  | 294 | 1.00 | ref |
|  | CAGE ≥ 2 | 157 |  | 12 | 1.91 | (1.01-3.60) |  | 46 | 1.06 | (0.75-1.51) |  | 35 | 1.69 | (1.13-2.52) |
|  | total | 4000 |  |  |  |  |  |  |  |  |  |  |  |  |
| Social participation | |  |  |  |  |  |  |  |  |  |  |  |  |  |
|  | participates | 1801 |  | 112 | 1.00 | ref |  | 394 | 1.00 | ref |  | 164 | 1.00 | ref |
|  | seldom, never | 1439 |  | 100 | 1.12 | (0.84-1.50) |  | 532 | 1.45 | (1.24-1.69) |  | 225 | 1.40 | (1.12-1.75) |
|  | total | 4767 |  |  |  |  |  |  |  |  |  |  |  |  |
| Sleep duration | |  |  |  |  |  |  |  |  |  |  |  |  |  |
|  | 7-9 hours | 2837 |  | 172 | 1.00 | ref |  | 786 | 1.00 | ref |  | 331 | 1.00 | ref |
|  | ≤ 6 or ≥ 10 hours | 376 |  | 37 | 1.40 | (0.96-2.05) |  | 125 | 1.03 | (0.83-1.29) |  | 54 | 1.08 | (0.79-1.48) |
|  | total | 4718 |  |  |  |  |  |  |  |  |  |  |  |  |
| *Adjusted for age, sex, education, marital status and chronic illness | | | | | | | | | |  |  |  |  |  |
| n varies from 4000 to 5351 due to different missing on the lifestyle-variables | | | | | | | | |  |  |  |  |  |  |
| Abbreviations used in the table: CAGE = screening questionnaire for risky alcohol consumption, CI = Confidence interval, HUNT = the Nord-Trøndelag Health Study, OR = Odds Ratio | | | | | | | | | | | | | | |
