# Supplementary material for: Prevalence, clustering and combined effects of lifestyle behaviours and their association with health after retirement age in a prospective cohort study, the Nord-Trøndelag Health Study, Norway
Source: BMC Public Health. 2020 Jun 10;20:900. doi: 10.1186/s12889-020-08993-y (PMC7288686; doi:10.1186/s12889-020-08993-y)
Supplement: Supplementary file 10 — Additional file 10. Lifestyle risk behaviours (HUNT2, 1995–97) and odds ratios (OR) for competing outcomes (HUNT3), multinomial logistic regression analyses.* [file 12889_2020_8993_MOESM10_ESM.docx]

| **Additional file 10.** Lifestyle risk behaviours (HUNT2, 1995-97) and odds ratios (OR) for competing outcomes (HUNT3), multinomial logistic regression analyses* | | | | | | | | | | | | | | |
| --- | --- | --- | --- | --- | --- | --- | --- | --- | --- | --- | --- | --- | --- | --- |
|  |  | No depression |  | Depression | | |  | Non-participation HUNT3 | | |  | Mortality during follow-up | | |
|  |  | n |  | n | OR | 95% CI |  | n | OR | 95% CI |  | n | OR | 95% CI |
| Daily smoking | |  |  |  |  |  |  |  |  |  |  |  |  |  |
|  | no | 3081 |  | 219 | 1.00 | ref |  | 837 | 1.00 | ref |  | 291 | 1.00 | ref |
|  | yes | 863 |  | 76 | 1.20 | (0.91-1.58) |  | 551 | 2.16 | (1.88-2.47) |  | 212 | 2.47 | (2.03-3.01) |
|  | total | 6130 |  |  |  |  |  |  |  |  |  |  |  |  |
| Physical activity | |  |  |  |  |  |  |  |  |  |  |  |  |  |
|  | active | 2165 |  | 131 | 1.00 | ref |  | 640 | 1.00 | ref |  | 233 | 1.00 | ref |
|  | inactive | 1554 |  | 143 | 1.44 | (1.12-1.85) |  | 621 | 1.29 | (1.13-1.48) |  | 223 | 1.37 | (1.12-1.68) |
|  | total | 5710 |  |  |  |  |  |  |  |  |  |  |  |  |
| Sitting time | |  |  |  |  |  |  |  |  |  |  |  |  |  |
|  | ≤ 7 hours | 2292 |  | 177 | 1.00 | ref |  | 663 | 1.00 | ref |  | 244 | 1.00 | ref |
|  | ≥ 8 hours | 1141 |  | 82 | 1.01 | (0.77-1.33) |  | 331 | 1.10 | (0.94-1.29) |  | 148 | 1.26 | (1.01-1.58) |
|  | total | 5078 |  |  |  |  |  |  |  |  |  |  |  |  |
| Alcohol | |  |  |  |  |  |  |  |  |  |  |  |  |  |
|  | CAGE ≤ 1 | 2882 |  | 206 | 1.00 | ref |  | 835 | 1.00 | ref |  | 319 | 1.00 | ref |
|  | CAGE ≥ 2 | 167 |  | 18 | 1.60 | (0.95-2.70) |  | 55 | 1.08 | (0.78-1.49) |  | 39 | 1.79 | (1.22-2.62) |
|  | total | 4521 |  |  |  |  |  |  |  |  |  |  |  |  |
| Social participation | |  |  |  |  |  |  |  |  |  |  |  |  |  |
|  | participates | 2028 |  | 137 | 1.00 | ref |  | 469 | 1.00 | ref |  | 180 | 1.00 | ref |
|  | seldom, never | 1602 |  | 136 | 1.17 | (0.91-1.51) |  | 636 | 1.44 | (1.25-1.66) |  | 243 | 1.37 | (1.10-1.69) |
|  | total | 5431 |  |  |  |  |  |  |  |  |  |  |  |  |
| Sleep duration | |  |  |  |  |  |  |  |  |  |  |  |  |  |
|  | 7-9 hours | 3171 |  | 231 | 1.00 | ref |  | 936 | 1.00 | ref |  | 355 | 1.00 | ref |
|  | ≤ 6 or ≥ 10 hours | 428 |  | 42 | 1.24 | (0.87-1.75) |  | 155 | 1.04 | (0.85-1.28) |  | 62 | 1.14 | (0.85-1.53) |
|  | total | 5380 |  |  |  |  |  |  |  |  |  |  |  |  |
| *Adjusted for age, sex, education, marital status and chronic illness | | | | | | | | |  |  |  |  |  |  |
| n varies from 4521 to 6130 due to different amount of missing on the lifestyle variables | | | | | | | | | |  |  |  |  |  |
| Abbreviations used in the table: CAGE = screening questionnaire for risky alcohol consumption, CI = Confidence interval, HUNT = the Nord-Trøndelag Health Study, OR = Odds Ratio | | | | | | | | | | | | | | |
